# Pain and Polypharmacy Diminish with Local Treatment of Mesenchymal Stem Cells Following Systemic Modulation of Inflammation: A Case Regarding Diabetic Foot Ulcers

**DOI:** 10.3390/cimb48010024

**Published:** 2025-12-25

**Authors:** Sara Marbelodeth Sosa Delgado, Juan Luis Amaya Espinoza, Jose Jesús Perez Correa, Brayan Andres Sandoval Pineda, Gisela Gutiérrez Iglesias

**Affiliations:** 1 Seccion de Estudios de Posgrado e Investigación, Escuela Superior de Medicina, Instituto Politécnico Nacional, Ciudad de México 11340, Mexico; ssosad@ipn.mx (S.M.S.D.);; 2 Laboratorio de Medicina Regenerativa y Estudios en Cáncer, Escuela Superior de Medicina, Instituto Politécnico Nacional, Ciudad de México 11340, Mexico; jamayae2500@alumno.ipn.mx (J.L.A.E.);; 3 Escuela Nacional de Ciencias Biológicas, Instituto Politécnico Nacional, Ciudad de México 11340, Mexico

**Keywords:** mesenchymal stem cells, pain, inflammatory molecules

## Abstract

Diabetic foot ulcers (DFUs) represent 6.3% of the various complications of type 2 diabetes mellitus, with a risk of development of up to 34%. Several factors contribute to the formation of ulcers, which are very difficult to treat as they hinder efficient wound healing. Patients experience persistent pain, which leads to the consumption of various medications (polypharmacy) due to the lesions not resolving. Conversely, this can increase the risk of various factors, including a chronic inflammatory state, which hinders the body’s own regenerative processes. Until now, treatment options have been limited to washing the wound and stimulating new tissue growth, but this is a painful and unsuccessful process. One of the treatment options is therefore cell therapy with mesenchymal stem cells, which have immunomodulatory characteristics and allow tissue regeneration, although the effect directly in pain is not totally clear. We have previously reported in our working group that patients with ulcers treated with mesenchymal stem cells (MSCs) have been able to integrate into their daily lives, although the pain related to the inflammatory state and polypharmacy has not been studied. Objective: This study investigates how the local administration of MSCs improves the condition of an ulcer by inducing tissue regeneration. It also shows how the concentration of systemic inflammatory biomarkers is modified in direct correlation with pain and the consumption of medications over time. Methods: Local administration of MSCs at 7 and 14 days, measuring pro- and anti-inflammatory cytokines relative to the healthy control group, evaluating wound healing, and monitoring the medications taken by the patient in conjunction with pain perception. Results: Cell administration showed that inflammatory molecules were reduced and anti-inflammatory molecules increased. This is reflected in the consumption of Non-Steroidal Anti-Inflammatory Drugs (NSAIDs) in relation to wound improvement, with a decrease in pain medication consumption of less than 50%. We provide evidence that locally administered mesenchymal stem cells influence systemic inflammatory processes necessary for tissue recovery, impacting patients’ polypharmacy consumption due to reduced perceived pain. Conclusions: This report establishes a direct link between mesenchymal stem cells and pain relief in type 2 diabetes ulcers, potentially paving the way for new pain therapies.

## 1. Introduction

Each year, approximately 18.6 million people worldwide suffer from Diabetic foot ulcers (DFUs). This complication significantly impacts patients with diabetes, 19% to 34% of whom will develop an ulcer during their lifetime. This is an alarming statistic, given that these lesions precede 80% of lower limb amputations [1,2]. DFUs are skin lesions on the extremities that greatly affect those who have them due to continuous pain. There is no effective treatment to reverse the lesions caused by this health condition. The chronic pain associated with these lesions is due to the involvement of peripheral sensory nerves [3], as well as an increase in the production of pro-inflammatory cytokines, such as Interferon gamma (INF-γ), Interleukin-6 (IL-6), and tumor necrosis factor alpha (TNF-α) [4,5].

In addition to causing physical disability, ulcer injuries cause pain, social isolation [6], sleep disturbances, behavioral changes, depression, and high hospital costs [7,8]. These biopsychosocial factors can have a significant negative impact on pain management, causing functional impairment, which leads to a loss of quality of life [9,10,11]. Currently, there are no treatments that effectively reduce pain and allow the affected tissues to regenerate.

Therefore, in response to this pain problem, patients, predominantly older adults, resort to taking Non-Steroidal Anti-Inflammatory Drugs (NSAIDs) without a prescription or increasing the recommended doses to cope with the pain. This leads each patient to consume more than five medications, resulting in polypharmacy, increasing the risk of health deterioration due to drug interactions, and even leading to frequent hospitalizations [12,13]. In the context of wound closure, various alternative therapies are based on administering mesenchymal stem cells (MSCs). The use of these cells has been proposed as an effective therapeutic strategy for managing DFU pain, thanks to their angiogenic, anti-inflammatory, and neuroprotective effects.

One study found that autologous transplantation of bone marrow-derived mesenchymal stem cells (BMSCs) in patients with diabetic foot and critical ischemia was associated with a significant reduction in ischemic pain [14]. This reduction was due to an increase in the distance or time of pain-free walking and a decrease in pain at rest. Notably, MSCs secrete proangiogenic and neurotrophic factors that promote microcirculation and neural integrity, suggesting an indirect benefit to other types of pain. Another research [15] addresses the relationship between MSCs and pain by reviewing the pharmacological treatment of neuropathic pain and advanced therapies. They point out that, while pharmacological control with pregabalin, duloxetine, tapentadol, and tricyclic antidepressants relieves neuropathic pain without reversing structural nerve damage, adipose tissue-derived MSCs represent a promising alternative for addressing the root cause of the problem. The authors mainly describe preclinical evidence in which these MSCs exert anti-inflammatory, proangiogenic, and neuroprotective effects on peripheral nerves in models of diabetic neuropathy. This translates into improved functional parameters and a potential reduction in neuropathic pain. However, they acknowledge that robust clinical trials confirming the direct impact of MSCs on neuropathic pain in humans are still lacking.

Our team has observed and reported that wounds can close in a short time by administering cells without the need for operating rooms or special facilities, which optimizes healthcare costs and represents a viable option for treating these complications arising from diabetes [16,17]. Our working group has previously reported that local administration of MSCs counteracts the inflammatory state of ulcers, which helps to stop damage, promote healing, and improve the patient’s overall condition within a few weeks. This has been explained by authors as being due to the fact that MSCs produce the anti-inflammatory cytokines Interleukin-4 (IL-4), Interleukin-10 (IL-10), and Interleukin-13 (IL-13), which suppress macrophage activation [18], improving the symptoms of ulcers and even restoring the functionality of the affected tissue.

Despite knowing the above, it is completely unknown whether the local administration of stem cells has a systemic anti-inflammatory effect in addition to an anesthetic effect in a patient with chronic ulcers, which would describe that, at the same time as the wound is closed, there is an impact on the patient’s polypharmacy status and recovery of quality of life.

Here we present the case of an elderly patient with a 10-year limb ulcer previously treated only with conventional therapy and polypharmacy for chronic pain. After receiving cell therapy, the ulcer healed completely. During the process, NSAID use was quantified according to pain perception and correlated with serum levels of key inflammatory molecules before and after MSCs treatment. This case also offers a detailed explanation of the mechanisms through which MSCs promote ulcer healing and modulate pain—an aspect not previously documented in cell-based therapies. Additionally, it provides an opportunity to evaluate the efficacy of MSCs as a therapeutic strategy for diabetic ulcers by comparing pro- and anti-inflammatory cytokine levels, reductions in drug consumption, and changes in pain perception before and after treatment.

## 2. Materials and Methods

### 2.1. Case Report

The patient, a 69-year-old woman, presented with a chronic ulcer measuring 17 cm in diameter on the lower extremity that had been in existence for a decade. The patient’s initial treatment regimen involved conventional therapy, with pain management being achieved through the high consumption of palliative medications (polypharmacy). Pursuant to the prior written consent of the patient, two doses of MSCs were administered on the first day of the evaluation and on the seventh day. A comprehensive evaluation was conducted to ascertain the concentrations of peripheral blood cytokines both prior to and following the conclusion of the treatment regimen. Concurrently, the wound closure process was meticulously monitored, and pain perception was assessed. The consumption of medications known as polypharmacy was subsequently evaluated using these findings.

### 2.2. MSCs Cell Culture

MSCs were obtained from the cell bank of the Laboratory of Regenerative Medicine and Cancer Studies, Escuela Superior de Medicina, Instituto Politécnico Nacional (IPN, Mexico City, Mexico), where they had been previously isolated from human umbilical cord tissue and cryopreserved. Umbilical cords used as the original cell source met the selection criteria of the Umbilical Cord Blood Bank of the National Medical Center “La Raza” (Mexico City, Mexico) and were handled in accordance with Mexican Official Standard NOM-253-SSA-2012 and the General Health Law. Molecular characterization of MSCs was performed according to the criteria of the International Society for Cell Therapy (ISCT) [19]. Cell population characterization was conducted prior to application using flow cytometry and PCR, and the expression of the analyzed molecular markers is summarized in Table 1.

For the present study, banked MSCs were expanded and cultured in DMEM/F12 medium (Gibco™, Thermo Fisher Scientific, Waltham, MA, USA) supplemented with 10% fetal bovine serum (FBS, Gibco™, Thermo Fisher Scientific, Waltham, MA, USA) at 37 °C in a humidified atmosphere with 5% CO_2_. Cell handling, processing, and expansion were performed in institutional facilities operating under biosafety level II conditions, following documented procedures aligned with Good Manufacturing Practice (GMP) principles and institutional regulatory frameworks.

### 2.3. Valuation and Treatment of Wound

The application of MSCs was performed by suspending them in a sterile isotonic saline solution and administering intradermally at both the wound edges and the wound bed. A standardized dose of 1 × 10^5^ ± 1 × 10^4^ cells per cm^2^ of wound area was applied following thorough cleansing and disinfection of the lesion. The number of administered cells was calculated based on the total wound surface area, using a defined cell concentration per square centimeter, a methodology previously reported and validated by our research group [20]. Previous studies from our team have demonstrated that this dosing strategy yields consistent and reproducible outcomes, supporting the robustness of the therapeutic regimen employed.

After cell administration, the wound was managed with sterile dressings and monitored on a daily basis. A second MSCs application was performed after a 7-day interval, and all relevant evaluations were conducted 14 days after completion of the treatment protocol. Wound healing was assessed by visual clinical inspection, with progression defined by the presence of proliferative tissue and/or epithelialization.

### 2.4. Pain Evaluation and Polypharmacy

**Figure 1:**
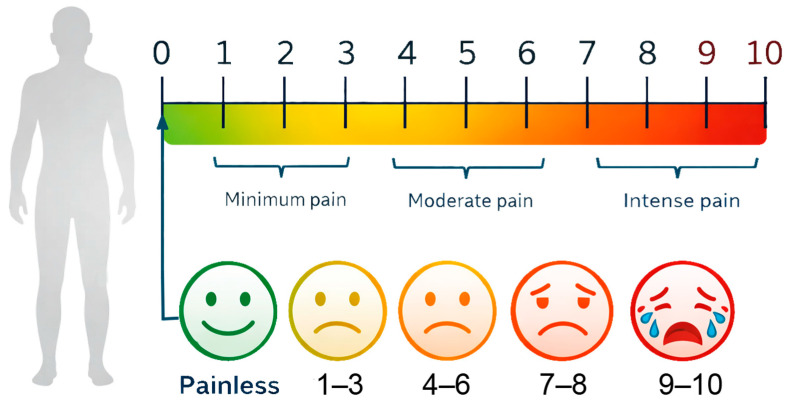
Verbal numerical scale (VNS) where the patient rates their pain from 0 to 10, and each numerical value on this scale correlates with the perceived intensity, allowing for standardized quantification.

The patient was consulted directly by questioning about their pain condition related to their general state. To assess the nociceptive condition and their response to treatment, at each appointment, patients were asked to quantify their pain perception using the verbal numerical scale (VNS) [21] from 0 to 10 (0 = no pain and 10 = maximum perceived pain sensation) and the values in points were considered in the interpretation of the data as shown in Figure 1.

We also delved into the disabling condition, the permissiveness or recovery of movement of the wound area and surrounding areas, and its evolution with respect to treatment. For a more complete understanding of pain coping, information was collected on the medications, variety, and doses that the patients reported consuming daily on their own initiative, which could be an indicator of their threshold and the intensity of their discomfort.

A comprehensive data set was also collected, encompassing the disabling condition, the permissiveness or recovery of movement in the wound area and surrounding regions, and its evolution with respect to treatment. In order to achieve a more comprehensive understanding of pain management, a comprehensive data collection process was initiated. This process entailed the compilation of information regarding the types and quantities of pharmaceutical medications consumed by the subjects. The data collected encompassed both the dosage and the frequency of drug intake over the course of a 24 h period. The subjects administered the medication on their own initiative. This was considered polypharmacy according to the threshold and the intensity of their discomfort.

### 2.5. Cytokine Analysis on Serum of Peripheral Blood

A peripheral blood sample of 3 mL was obtained from a 69-year-old female patient prior to the start of treatment using MSCs, and this was considered the baseline control. For the negative control, a blood sample of the same volume (3 mL) was collected from a healthy 39-year-old male volunteer. A second 3 mL peripheral blood sample was obtained from the patient at the end of treatment on day 14 for comparative analysis. Serum was separated by means of centrifugation at 1500× *g* for 15 min, following which it was stored at −80 °C until use. The detection of cytokine levels in serum samples was accomplished through the utilization of the BD FACSAria™ Fusion flow cytometer (Becton, Dickinson and Company, San Jose, CA, USA), available at the Escuela Superior de Medicina, Instituto Politécnico Nacional (ESM-IPN, Mexico City, Mexico), employing the Cytometric Bead Array (CBA) Human Th1/Th2 Cytokine Kit (BD Biosciences, San Jose, CA, USA; Catalog No. 551809). The flow cytometer was calibrated using cytometer configuration beads, and the assay was subsequently performed. The data underwent a Two-Way Analysis of Variance (ANOVA) in GraphPad Software Inc. (San Diego, CA, USA).

## 3. Results

### 3.1. Wound Assessment

**Figure 2:**
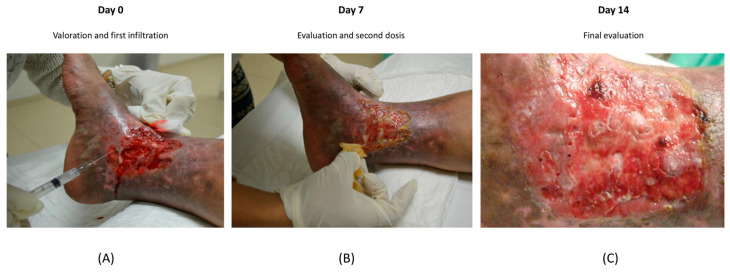
MSCs treatment and its evolution until final evaluation. The lesion is shown on day 0 (**A**) and its infiltration as described in Materials and Methods. Subsequently, on day 7 of the first infiltration (**B**), tissue improvement was observed until day 14 (**C**), where pink tissue recovery with closure of the lesion was observed.

On the initial day of the experiment (designated as “day zero” herein), the first MSCs infiltration procedure was conducted. As evidenced in Figure 2A, the wound was meticulously cleansed, and the cells were administered in accordance with the protocols outlined in the Materials and Methods Section. On the seventh day, the lesion was examined to determine the extent of cell proliferation and the presence of new tissue formation, which was indicated by the presence of yellow tissue. Subsequently, on day 14 (see Figure 2B), the final assessment was performed, showing wound closure with epithelialization (pink tissue) and the appearance of scabs as a result of wound healing. A decrease in the area and depth of the wound was observed on day 7, and the dark color surrounding the ulcer persisted on day 7 but improved on day 14 (Figure 2C), resulting in a healthy pink appearance.

### 3.2. Pain Perception Along Treatment

The effect of MSCs on pain response was a primary focus of the study, given its role in regulating pain, function, and mobility. However, given the inability to conduct anti-nociceptive experiments on patients, a direct questioning method was employed to assess their perception of pain. This method utilized a scale ranging from 0 to 10 points, where 0 represented the absence of pain and 10 represented the maximum perceived intensity of pain. Accordingly, cell therapy resulted in a significant decrease in pain perception, with discomfort levels being reduced to only 20% of the initial level (see Table 2 for further details). Therefore, a decrease in pain of more than 50% was observed seven days after the initial treatment, with the pain level reduced by four points on a scale of ten. Furthermore, within 14 days of the initial treatment and seven days after the second treatment, an 80% reduction in pain perception was noted, as indicated by a single cross in Table 2.

### 3.3. Evaluation of Polypharmacy

**Figure 3:**
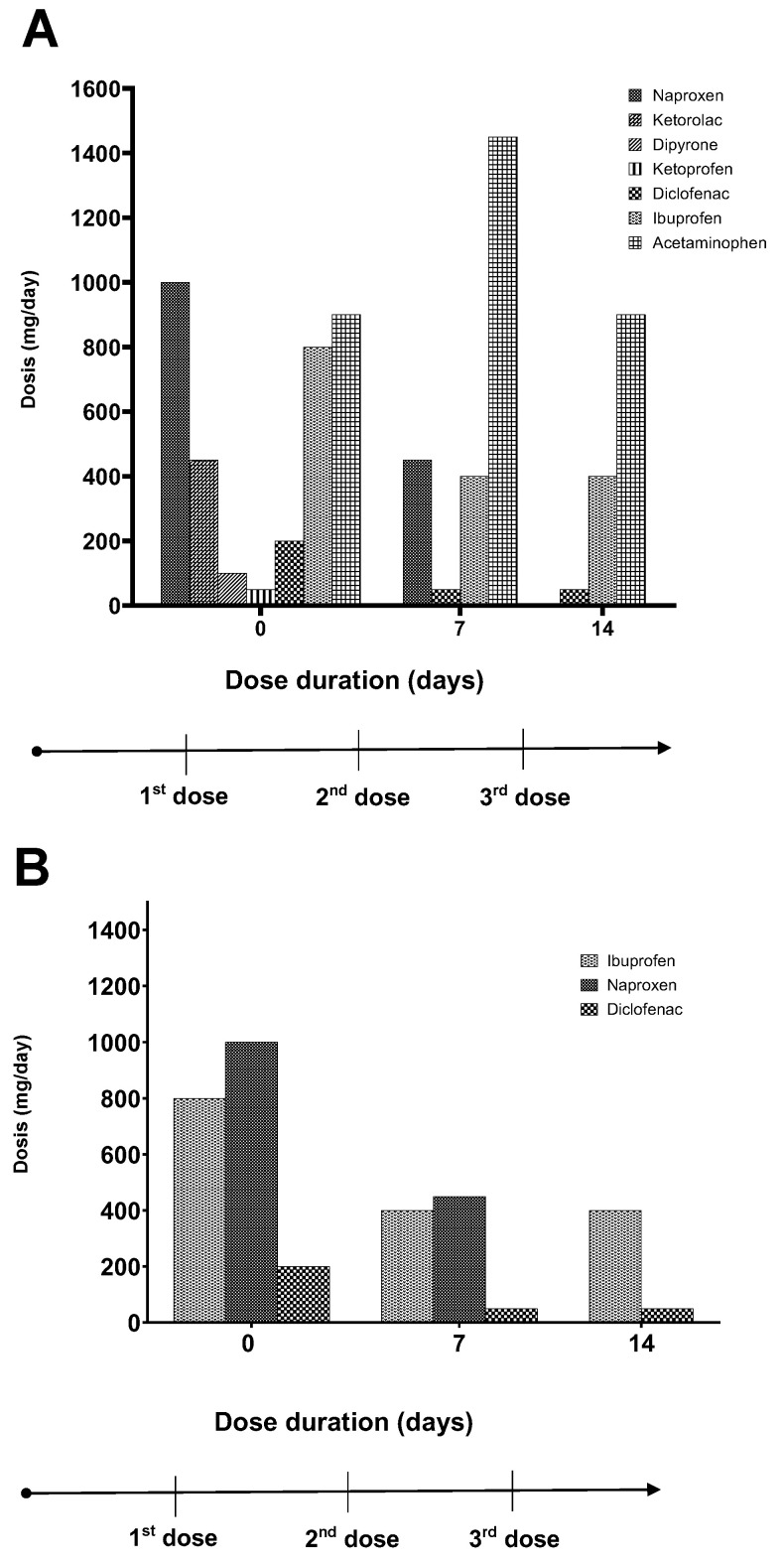
Consumption of medications in mg/day by the patient. (**A**) All medications used when MSCs were administered on day 0 (the first dose), at day 7 (day of the second dose), or on the day of final analysis (day 14). (**B**) Special focus on naproxen, ibuprofen, and diclofenac consumed per day.

Additionally, due to the pain that the patient experienced, the nonsteroidal or anti-inflammatory medications that he had taken daily were registered (Figure 3). Before treatment, the pain was controlled (although not totally) using a combined range of seven medications with different pharmacological potencies consumed over one day (Figure 3A). After 7 days from the first application of MSCs, the pain decreased, and it was reduced by the variety of medicines taken, using only four commonly used ones from the original seven; these freely accessible medicines had moderate pharmacological potency (acetaminophen, ibuprofen, and diclofenac). On day 14, the consumption of medications was reduced to just three of them acetaminophen, ibuprofen, and diclofenac. We had a special interest in comparing three accessible and specific medications (naproxen, ibuprofen, and diclofenac) because the taken doses decreased with each cell application (Figure 3B) until the patient eventually recovered their mechanic function and was capable of movement; due to the pain tolerance and because the wound healing allowed soft contact with their clothes, their lifestyle improved to near-normal. It is necessary to complete studies, ones that provide supporting information on these effects with biomechanic probes, histology, immunochemical, and tissue regeneration, to describe, in more detail, what mechanisms are implicated in regenerative processes.

### 3.4. Localized Administration of MSCs Modified Systemic Pro-Inflammatory and Anti-Inflammatories Molecules

**Figure 4:**
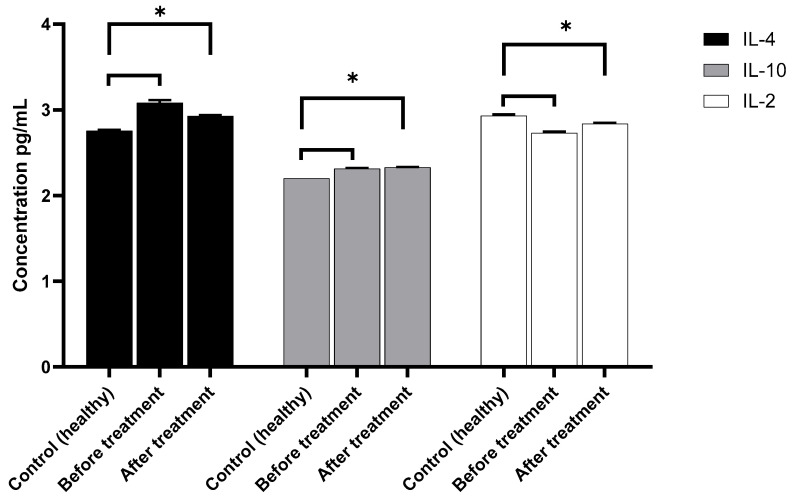
Anti-inflammatory cytokine serum concentrations (pg/mL): IL-4, IL-10, and IL-2. Values are shown for healthy subjects (control group), patients before treatment, and patients after treatment with MSCs. Significant increases in anti-inflammatory cytokine concentrations were observed after therapy compared to baseline values. Statistical differences are indicated with an asterisk (*), *p* = 0.01.

Inflammatory and anti-inflammatory cytokines were quantified in the serum of the patient; these molecules reflect the immunologic state of the patient. The major aim of this evaluation was to determine if local administration of MSCs could impact not only in inflammatory state of the wound, either at the general level (Figure 4).

Results showed that anti-inflammatory cytokine IL-4 had a higher level at the start of the treatment, but at the final treatment, IL-4 diminished, and it was not at a healthy level (Figure 4). IL-10 showed slight augmentation in both conditions (day 0 and 14) compared with the healthy control; meanwhile, the concentration of IL-2 stayed on day 0 lower than the control but finished with a slight tendency to increase on day 14 (final evaluation).

**Figure 5:**
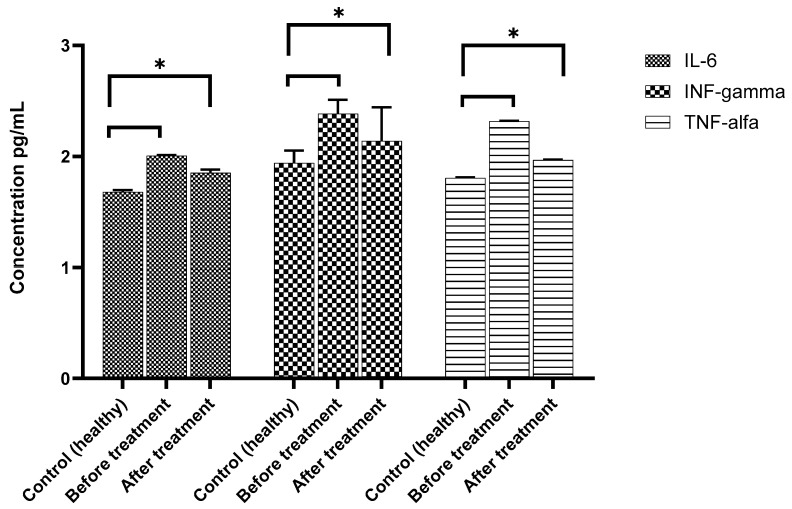
Serum concentrations of proinflammatory cytokines IL-6, IFN-γ, and TNF-α (pg/mL). Values are compared in healthy subjects (control), patients before treatment, and patients after treatment with mesenchymal stem cells. Proinflammatory levels showed a significant decrease after therapy compared to the patients’ baseline status. Statistical differences are indicated with an asterisk (*) *p* ≤ 0.05.

When we analyzed proinflammatory cytokines (Figure 5), we observed that IL-6 levels were higher than the control at the beginning of treatment, and after 14 days from the first dose, they diminished; however, the levels did achieve healthy concentrations, and the performance was remarkably reduced. Serum levels of IFγ remained elevated with similar concentrations in both measurements (day 0 and 14), always higher than the control (Figure 5), although on day 14, the IFNγ levels decreased slightly. Interestingly, in the case of the inflammatory molecule TNF-α, similar to IL-6, it was drastically augmented at the beginning of treatment, but at day 14, concentrations were markedly low, falling between the midpoint of the healthy levels compared with the day of the first dose of MSCs.

## 4. Discussion

The results demonstrated that local administration of MSCs resulted in a progressive regenerative effect, so in the medical evaluation of the lesion, it was observed that the treatment induced the natural debridement process, evident from day 7 until day 14 of the final assessment, when complete wound closure and a reduction in tissue swelling were observed, allowing tissue regeneration as expected based in other work [22]. As demonstrated in the extant literature, ulceration is challenging to remediate with traditional therapeutic modalities due to the prolonged duration and protracted nature of recovery. A review of the relevant literature reveals that the recovery of healthy tissue following ulceration can take a minimum of two months [23,24]. In certain cases, ulceration may not result in regeneration of the affected tissue [22]. Therefore, the evaluation of tissue recovery was conducted with consideration for the subject’s pain and the overall inflammatory state.

The monitoring of the lesion during treatment was accompanied by the documentation of the patient’s pain. This data has a significant relevance, as pain is the primary clinical indicator that can be objectively measured and reflects the patient’s quality of life, which is a crucial factor in the management of most diseases [25,26]. In particular, ulcer pain in its open wound stages is characterized by its intensity, accompanied by irritation, inflammation, and the inability to move or wear clothing. Moreover, it poses a risk of infection due to inadequate pain management through the prolonged use of home remedies that maintain the affected area in a moist state [27,28]. The evaluation of pain following treatment revealed a significant improvement, with the pain record for day 7 of treatment decreasing by 50% and reaching a 75% reduction on day 14 (Figure 3). These findings suggest that the administration of MSCs not only enhances ulcer closure but also provides analgesia.

Conversely, the severity of pain has been directly associated with the ongoing inflammatory response. Medical opinion suggests that this should not be regarded as a universal principle. However, in the context of open ulcers, this principle does apply due to the condition of the skin, which is characterized by its irrigated state and the presence of sensory fibers. These fibers, in the presence of inflammation, induce persistent and constant pain [29,30]. In order to ascertain whether the patient’s pain was associated with a systemic inflammatory process, the presence of pro- and anti-inflammatory cytokines was evaluated. Following a 14-day period of treatment, a marked increase was observed in IL-2 levels, concurrent with a decline in IL-4.

Among the pro-inflammatory cytokines, IFN, IL-6, and TNFα, the latter two demonstrated a substantial decrease. The underlying mechanism by which MSCs facilitate wound healing may involve the restoration of a homeostatic environment within the wound, thereby enabling the secretion of molecules such as TNFα, IFNγ, and IL-6 have been described [31]. The expression of this molecule induces the attraction, migration, and infiltration of native platelets, fibroblasts, and keratinocytes that remodel the area of the injury. This phenomenon has been demonstrated in murine models of wound healing, where TNFα has been shown to stimulate mesenchymal cells, thereby accelerating the process of regeneration [32] and promoting angiogenesis, as in chronic lesions via VEGF production or as described for wounds treated with MSCs secretome, where polarization of the inflammatory macrophage to the M2 phenotype occurs [33].

This process precedes fibroblastic and granular tissue formation, and the new extracellular matrix provides cellular support to stimulate wound epithelialization and contraction, which indicates regeneration [15].

Finally, inflammation and pain perception influence the decision to take medication to alleviate discomfort in 71.4% of patients with chronic pain [34,35]. A review of the medication records revealed that, on day 7, the number of medications taken daily decreased from seven to four. By day 14, only acetaminophen, ibuprofen, and diclofenac were consumed. A comparison of the last three medications reveals a 50% decrease in dosage on day 7, with mild doses sufficient to manage pain on day 14. These findings suggest that mesenchymal stem cells may play a role in regulating inflammation, pain, and medication intake. This study integrates data on ulcer closure, pain, and CMM consumption, and it specifically describes the molecules that, at the systemic level, are indicators of the reduction in the inflammatory process reflected in the reduction in the patient’s pain. In situ wound treatments involving the use of mesenchymal stem cells (MSCs), as well as factors and cytokines such as tumor necrosis factor alpha (TNFα), interferon gamma (IFNγ), and interleukin-6 (IL-6), have been documented [4]. These factors play a crucial role in processes such as attraction, migration, and infiltration of native platelets, fibroblasts, and keratinocytes, which collectively contribute to the remodeling of the injury site. During the inflammatory phase of the healing process, macrophages and polymorphonuclear leukocytes play an important role, as they debride and remodel the affected area. These cells also release growth factors and cytokines to promote the proliferation phase and initiate angiogenesis. However, in cases where homeostasis is disrupted due to exacerbated inflammation, the tissue does not fully consolidate, thereby hindering the process of ulcer closure and resulting in a state of chronic inflammation [36].

The results demonstrated that IL-4 levels increased, while IL-2 levels tended to be elevated compared to healthy parameters. This phenomenon may be associated with the improvement in the ulcer’s appearance.

## 5. Conclusions

The findings of this investigation demonstrate that therapy based on the administration of mesenchymal stem cells (MSCs) significantly enhances the healing process of chronic ulcers. The intervention was designed to reduce the local inflammatory process by decreasing proinflammatory cytokines and improving the systemic immunological molecular profile. The result of this process was the establishment of a microenvironment with the capacity to induce alterations in macrophages, including the transition from the M1 to the M2 state. This transition is a critical component of the process of tissue repair. This immunological modulation was clinically correlated with the patient’s pain response to treatment. A progressive decrease in pain was induced, and the patient experienced enhanced pain management, as evidenced by a reduction in the number of pharmaceutical agents and doses administered. The patient’s quality of life exhibited a significant enhancement as a consequence of the healing process of the ulcer.

The present study is situated within the domain of comprehensive patient monitoring, wherein an association is posited between clinical progress and molecular alterations observed from day zero to day fourteen of treatment with mesenchymal stem cells. The integration of these two fields is imperative to provide a more precise and robust approach to understanding the mechanisms by which mesenchymal stem cells exert their benefits, not only on wound closure but also on the projection of systemic improvement in patients with local cell treatment. This treatment was able to reverse tissue damage, allowing ulcer closure and better pain management.

## Figures and Tables

**Table 1:** Molecular markers of MSCs analyzed by flow cytometry and RT-PCR.

Molecular Marker	Cellular Population (%) and SD	Relative RNA Expression (%)
CD90	97.4 ± 5.5	100 ± 15
CD73	97.4 ± 6.8	100 ± 18.2
CD105	36.0 ± 5.5	100 ± 20
HLA-DR	0.0	0.0
CD34	6.0 ± 0.15	0.0
CD45	0.2 ± 0.02	0.0

Molecular markers of MSCs used in the treatment of chronic ulcer, verified by flow cytometry and RT-PCR, as previously described. The percentage with standard deviation is shown.

**Table 2:** Changes in pain intensity during the course of treatment.

Time of Treatment	Pain Intensity
Day 0	+++++ (10 points)
Day 7	++ (4 points)
Day 14	+ (2 points)

Reduction in pain intensity during the course of treatment. Perception of pain intensity in relation to the day of treatment and its scale in points. The maximum pain perception level was established at 10 points with 5 crosses (representing the highest level of pain). The pain intensity was subsequently measured on days 7 and 14 following treatment.

## Data Availability

The data supporting the findings of this study are contained within the article.
